# Twenty Years of Passive Disease Surveillance of Roe Deer (*Capreolus capreolus*) in Slovenia

**DOI:** 10.3390/ani11020407

**Published:** 2021-02-05

**Authors:** Diana Žele Vengušt, Urška Kuhar, Klemen Jerina, Gorazd Vengušt

**Affiliations:** 1 Institute of Pathology, Wild Animals, Fish and Bees, Veterinary Faculty, University of Ljubljana, Gerbičeva 60, 1000 Ljubljana, Slovenia; diana.zelevengust@vf.uni-lj.si; 2 Institute of Microbiology and Parasitology, Veterinary Faculty, University of Ljubljana, Gerbičeva 60, 1000 Ljubljana, Slovenia; urska.kuhar@vf.uni-lj.si; 3 Department of Forestry and Renewable Forest Resources, Biotechnical Faculty, Večna pot 83, 1000 Ljubljana, Slovenia; klemen.jerina@bf.uni-lj.si

**Keywords:** disease surveillance, postmortem examination, roe deer, Slovenia, *Capreolus capreolus*

## Abstract

**Simple Summary:**

Wildlife can serve as a reservoir for highly contagious and deadly diseases, many of which are infectious to domestic animals and/or humans. Wildlife disease surveillance can be considered an essential tool to provide important information on the health status of the population and for the protection of human health. Between 2000 and 2019, examinations of 510 roe deer carcasses were conducted by comprehensive necropsy and other laboratory tests. In conclusion, the results of this research indicate a broad spectrum of roe deer diseases, but no identified disease can be considered a significant health threat to other wildlife species and/or to humans.

**Abstract:**

In this paper, we provide an overview of the causes of death of roe deer (*Capreolus capreolus*) diagnosed within the national passive health surveillance of roe deer in Slovenia. From 2000 to 2019, postmortem examinations of 510 free-ranging roe deer provided by hunters were conducted at the Veterinary Faculty, Slovenia. A comprehensive necropsy was performed. According to the results of the necropsy, the samples were subjected to microscopic, histopathological, bacteriological, parasitological, or virological examination. The most frequent causes of death in roe deer were infectious diseases (67%), followed by noninfectious diseases (28%). Of all deaths, parasitic infections represented 48%, bacterial infections 14.8%, trauma 12.5%, and metabolic disorders 9.8%. Less frequent causes were diseases like neoplasia and mycotic infections, winter starvation, hernias, and lightning strike. This study covered an estimated 1% of the total disease-related mortality of roe deer in Slovenia. Comparisons of sex/age structure indicated that hunters did not provide random samples (e.g., young males were disproportionately represented). Therefore, such monitoring does not ensure an unbiased assessment of the significance of the individual disease for the mortality of the population; however, it can provide credible evidence of whether or not a particular disease is present in a population. We show that no identified disease in roe deer in Slovenia can be considered a significant health threat to roe deer, other wildlife species, or humans.

## 1. Introduction

In recent decades, international attention to wildlife diseases, including surveillance and monitoring programs, has increased [1,2]. Wildlife diseases occur in numerous forms in a variety of animal species and populations around the globe. In addition, wildlife can serve as a reservoir for highly contagious and deadly diseases, many of which are infectious to domestic animals or humans, and may impact biodiversity [1,3] and the economy. Health monitoring and surveillance is an integral part of wildlife disease identification and management. Regular surveillance programs provide evidence of national disease-free status and confirm the disease-free status of major infectious diseases in free-ranging animal populations [4,5]. In general, health surveillance involves three crucial steps: prevention, control, and eradication when the existence and extent of pathogen have been established. Continuous disease control and management have shown that surveillance programs are of great benefit to public and animal heath [6,7].

Surveys based on postmortem examinations of carcasses can provide important information on the health status of the population, including age and sex structure and the causes of mortality [8]. Infectious diseases in roe deer can affect other wildlife, domestic animals, and, when zoonotic agents are involved, humans. Direct or indirect interactions also occur with domestic animals on pastures or at shared water sources [9] or with hunting dogs [7]. Therefore, the health status of roe deer can serve as a bioindicator of environmental health and is relevant to the health of other wildlife, domestic animals, and humans [7]. To date, reports of specific roe deer diseases have been scarce, with scientific work on the general health surveillance of roe deer in Europe has only been reported from Switzerland [7], Sweden [10], and France [11]. With an estimated population of 10 million, European roe deer is the most common and widespread deer species in Europe [12]. It is considered a woodland animal, although it also inhabits more open landscapes with woody structures such as hedgerows [13]. Due to its high adaptability to new habitats, it has successfully occupied the fragmented landscapes and has coped effectively with modern agricultural expansion [14]. In recent decades, the population numbers and at the same time the hunting bag of roe deer have greatly increased in most parts of Europe [15]. This makes roe deer one of the crucial game species and an important prey of large predators in Europe [12,16]. Therefore, continuous surveillance of the health status of roe deer is important both for the conservation of the species and for the health of other wildlife, domestic animals, and humans [7]. In Slovenia, about 80% of the area is permanently inhabited by roe deer [17]. With about 110,000 individuals, roe deer are one of the most important game species in the country [18].

The present study provides an overview of 20 years of diagnostic investigations of roe deer carcasses within the framework of a national passive wildlife health monitoring program in Slovenia.

## 2. Materials and Methods

### 2.1. Samples

**Figure 1:**
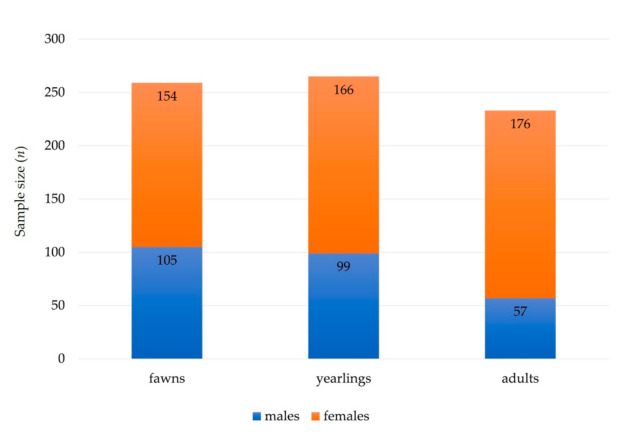
Summary of sex (males and females) and age (fawns, yearlings, and adults) structure of free ranging roe deer sampled and analyzed for diseases.

**Figure 2:**
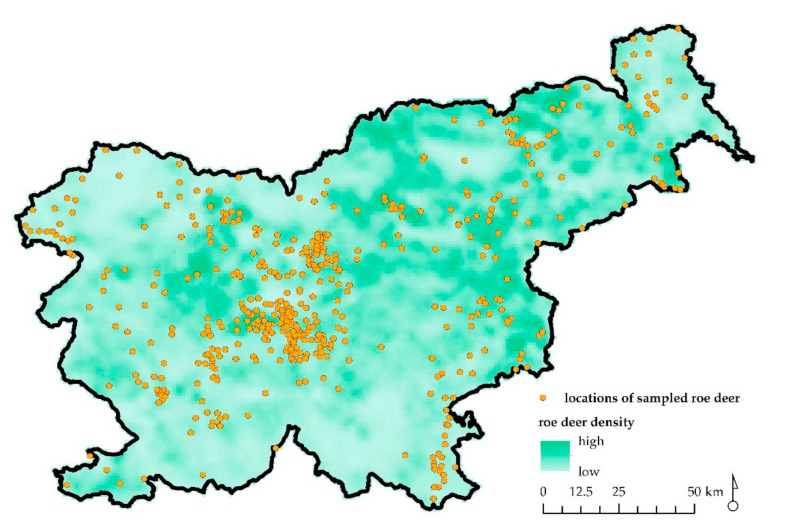
Map of Slovenia with depicted locations of sampled and analyzed free ranging roe deer (*n* = 510; orange dots) and roe deer relative population density (lowest to highest).

We collected complete records of 510 necropsies on roe deer carcasses (male, *n* = 248; female, *n* = 262) (Figure 1), obtained under a national wildlife passive health surveillance program in Slovenia in the period of 2000–2019 (Figure 2). Almost 64% (*n* = 326) of the samples were collected from roe deer found dead in the wild, 29% (*n* = 148) of the roe deer were legally shot due to observed signs of disease, and 7% (*n* = 36) of the animals were shot during the regular annual culling. Volunteer gamekeepers and professional game wardens from all over the country were encouraged to provide samples of roe deer through various information channels (hunter magazine and administrative services of hunter organizations). Carcasses of roe deer found dead in the wild, animals harvested due to signs of disease, or animals shot during the regular annual culling showing unusual health characteristics were submitted to the Veterinary Faculty, University of Ljubljana. The age of the animals was estimated by authorized committees during the mandatory annual inspection of hunted ungulates [19]. Eruption patterns and tooth wear were used to estimate the age of roe deer. The animals were then divided into three age groups: fawns (<1 year old), yearlings (1–2 years old), and adults (3+ years old) (Figure 1). The approval of the Ethics Committee/Welfare Authority was not required, as all samples were taken postmortem.

### 2.2. Laboratory Methods

Tissue samples collected at necropsy were fixed in 10% neutral buffered formalin, processed, embedded in paraffin, cut, and stained with hematoxylin and eosin (H&E), Periodic acid-Schiff staining method (PAS), Gram, and Ziehl–Neelsen, using standard protocols. If necessary, additional special staining was performed for tissue-based diagnosis.

For bacteriological examination, the culture from tissue samples was usually prepared for blood agar (5% sheep blood) and incubated aerobically and anaerobically at 37 °C. After 24 h incubation, the blood agar plates were examined for the presence of pathogenic bacteria. If necessary, the plates were incubated for another 48 h. The isolates were biochemically characterized using the API (commercial system API bioMerieux, Lyon, France) and later by MALDI-TOF-MS (matrix-assisted laser desorption ionization time-of-flight mass spectrometry) (Bruker Daltronik GmbH, Bremen, Germany) according to the manufacturer’s instructions. MALDI-TOF MS was introduced into the microbiological routine in the Veterinary Faculty in 2015. Since then, it has almost replaced biochemical identification. One of its main advantages is that bacterial identification using MALDI-TOF MS takes minutes.

Gastrointestinal tract, lung, liver, abdominal cavity, and skin were examined for the presence of parasites. The abomasum and small and large intestines were removed, cut open lengthwise, and the contents were washed through a series of sieves. Liver and lungs were cut into pieces and immersed in lukewarm physiological solution (0.9% NaCl solution). The helminths were removed from the intestine and other organs and fixed in 70% ethanol. Species differentiation was based upon the microscopic investigation of the morphology of the male nematodes as described by Soulsby [20], Niewiadomska [21], and Anderson et al. [22] after mounting in lactophenol. For the detection of Eimeria oocysts and helminths eggs, the feces samples were analyzed by the flotation technique with NaCl solution and sedimentation, as described by Eckert [23]. To diagnose lungworm larvae, we used the Baermann-Wetzel technique as described by Eckert [23]. *Sarcocystis* spp. were an incidental finding on histologic examination of cardiac or skeletal muscle and classified as a secondary finding. Formalin-fixed tissues were paraffin-embedded and 4 μm sections were prepared and stained with hematoxylin and eosin according to the standard procedures. The slides were than investigated under light microscope.

For the detection of papilloma viruses (PV) in skin neoplasias using PCR, the skin tissue samples were stored at −70 °C until testing. Ten percent suspensions of the tissue samples were prepared (1 cm^3^ of tissue was added to 9 mL of RPMI medium 1640 (Thermo Fisher Scientific, Carlsbad, CA, USA)). The suspensions were homogenized and centrifuged at 2000 × g for 10 min, and the supernatant was stored at −70 °C if not immediately processed. The supernatant was used for nucleic acid extraction with the DNeasy Blood & Tissue Kit according to the manufacturer’s instructions (Qiagen, Germany). PCR with a combination of primers (CanPVf: 5′-CTTCCTGAWCCTAAYMAKTTTGC-3′, FAP64: 5′-CCWATATCWVHCATITCICCATC-3′) already described by Lange et al. [24] amplifying a 383 bp-long fragment of the L1 gene was used for the detection of PVs [24,25]. The PCR products were subjected to electrophoresis in a 1.8% agarose gel.

### 2.3. Evaluation of Representativeness of Surveillance

To obtain an approximate estimate of the representativeness of passive sampling based on the voluntary provision of roe deer carcasses by hunters, we analyzed (1) the temporal trends in the annual frequency of the samples provided, (2) their spatial distribution, and (3) the approximate share of the analyzed samples in the total disease-related roe deer mortality in the country. This was achieved using data from National Wildlife Mortality Register [17], which must be maintained by all hunting ground managers as required. The registry contains data on the recorded total mortality of wildlife species, and for each individual, the data include sex, age, location of harvest, and the estimated cause of mortality (including the category “disease”). In Slovenia, the course material for a hunting examination includes basic chapters on wildlife diseases of the main game species, with a focus on the recognition of visual signs of disease. For statistical analysis, we used several methods/tests considering the purpose of the analysis and the limitations of the available data. The presence of systematic temporal changes in the number of roe deer provided for necropsy during the research period was analyzed using linear regression, trends in the temporal changes in proportions with nonparametric correlation, and differences in sex and age structure between the carcasses provided for examination and all recorded cases of natural mortality with tests of homogeneity of structures (Chi-squared statistics). All statistical analyses were performed with Statistica 10.0 (StatSoft, Inc., Tulsa, OK, USA).

## 3. Results

In 2000–2019, a total of 510 roe deer were collected from hunting grounds covering a large part of species home range in Slovenia. A total of 283 bacterial identifications, 398 parasitological, 334 pathohistological, and 11 PCR tests for PV detection were performed. The diagnoses of the primary cause of mortality and the causes associated with the main disease are listed in Table 1 and Table 2.

Death followed by extensive trauma occurred in 64 animals. These were the result of traffic accidents (*n* = 31), firearms (*n* = 16), predation (*n* = 8), or antler puncture wounds caused by other roe deer bucks (*n* = 9). The metabolic diseases described (*n* = 50) included metabolic acidosis (*n* = 29) with rumen dysfunction, bloat (*n* = 13), and plant poisoning (*n* = 8). In 19 cases, neoplasias of different origin were diagnosed. Fibropapillomatosis (n = 13) was the most frequently diagnosed tumor, followed by lymphosarcoma (*n* = 1), tibial osteosarcoma (*n* = 1), mandibular osteoma (*n* = 1), liver carcinoma (*n* = 1), thyroid carcinoma (*n* = 1), and histiocytoma cutis malignum (*n* = 1). Hernia (inguinal hernia, *n* = 2; and umbilical hernia, *n* = 1), winter starvation, lightning strike, and others (dystocia) were further noninfectious causes of roe deer diseases.

The reported infections were mainly attributed to parasitic infections, which were diagnosed in 246 cases. An overview of the parasitic species found in roe deer is listed in Table 2. The majority (76.4%) of the infected roe deer in the present study harbored several helminth species. Nematodes were the predominant group of helminths (*Haemonchus contortus*, *Trichuris capreoli*, *T. ovis*, *Spiculopteragia asymmetrica*, *Skrjabinagia kolchida*, *Chabertia ovina*, *Trichostrongylus axei*, *T. capricola*, *T. colubriformis*, *Oesophagostomum venulosum*, *O. radiatum*, *Ostertagia leptospicularis*, *O. ostertagi*, *O. circumcincta*, *Cooperia* spp., *Capillaria bovis*, all in the intestine; *Muellerius capillaris*, *Neostrongylus* spp., *Dictyocaulus viviparus*, all in the lung; *Setaria* spp. in the abdominal cavity; and *Onchocerca* spp. in the skin). The cestodes included *Taenia hydatigena*, *T. krabbei* cysticercosis, *Moniezia benedeni*, *and M. expansa*; trematodes included *Fasciola hepatica* and *Dicrocoelium dendriticum*; and protozoa included *Eimeria* spp. and *Sarcocystis* spp. The ectoparasite fauna included three species of hippoboscid fly (*Lipoptena cervi*, *Hypoderma diana*, and *Cephenemyia* spp.), a tick (*Ixodes ricinus*), and a louse (*Damalinia meyeri*).

Bacteria were responsible for 75 infections. The reported infections were mainly attributed to 12 microorganisms (Table 2). Within the mixed flora *Klebsiella pneumoniae*, *Morganella morganii*, *Providencia alcalifaciens*, *Nocardia farcinica*, and *Hafnia alvei* were sporadically isolated.

*Aspergillus fumigatus* was the only confirmed mycotic infection categorized as the cause of death in six animals.

In the period studied, the average total annual mortality (harvest is the predominant cause, followed by vehicle collisions, etc.) of roe deer in Slovenia was 43,621 animals (CI 95%: 43,226–44,015). The number of registered roe deer that died presumably due to diseases based on visual determination of the bodies by hunters was 542 (CI 95%: 374–710). There were 998 additional mortality cases (CI 95%: 806–1190) reported on average annually for which the hunters were unable to determine the cause of death themselves, a significant proportion of which were probably due to disease. Assuming that approximately one-quarter of all-natural mortality is recorded, as shown by earlier studies in Slovenia, the actual annual roe deer mortality is about 2200 (approx. 542 × 4), but probably closer to 6200 (approx. 4 × (542 + 998)). During the same period, hunters provided the samples (whole carcasses or organs) of 25.5 (CI 95%: 21–30) animals on average, which indicated that our study covers only about 1% and probably 0.5% of the total disease-related roe deer mortality in Slovenia.

Annually, 13–52 animals were provided by hunters for examinations, but this number increased during the research period (β = 0.83, *p* < 0.01) and averaged 15 animals per year in the first 3 years and 41 animals in the last 3 years. As the size of the roe deer population in Slovenia has been gradually decreasing over the last decades, the sampling intensity increased in absolute and relative terms.

The roe deer submitted for examination origin from 167 of the 411 hunting grounds in Slovenia and from 5 of the total 9 special purpose state hunting grounds. The hunting grounds from which the samples were collected cover about 40% of the total Slovenian territory and over 50% of the total Slovenian territory with permanent presence and reproduction of roe deer. The samples were mostly delivered from hunting grounds where roe deer density is high, which indicated that the sampling covered practically the largest part of the core roe deer habitat in the country.

The age and sex structure of the samples supplied differed from the structure of all the recorded animals whose mortality was identified by the hunters as a disease. Among the samples provided, the proportion of males (51% vs. 32%) and young animals/juveniles (36% vs. 20%) was higher than in all recorded cases of disease-related mortality; male young animals were strongly over-represented in the samples provided (21% of all laboratory samples vs. 8% in all recorded mortality cases). The difference in structure thus indicates that the samples provided were not random but the result of a specific selection by the hunting ground managers.

## 4. Discussion

Roe deer are one of the most widespread free-living ungulates and an important game species in Slovenia with about 110,000 individuals [18]. The yearly hunting bag in 2019 was 31,856 animals [26]. According to the Slovenian hunting authorities, several hundred animals die from other causes (i.e., between 4000 and 6000 roe deer are killed on roads every year) [27], including diseases.

This is the first comprehensive report on the causes of deer deaths in Slovenia based on passive surveillance. Passive wildlife health surveillance aims to detect the presence or spread of diseases or infections or the early detection of new emerging or re-emerging diseases in a country and can provide valuable information for national surveillance systems. Further data for surveillance; monitoring; notification of diseases, infections, and infestations; and the provision of epidemiological information are defined in the Office International des Epizooties (OIE) [28].

According to Akdesir et al. [29], wildlife carcasses submitted for necropsy are generally not representative of the entire population and country due to numerous factors influencing the submission of carcasses. The data presented show that in Slovenia, similar to Switzerland [7] and Sweden [10], only a small proportion of carcasses are submitted annually for diagnostic examination. Nevertheless, wildlife health surveillance is a valuable source of information on the causes of mortality, susceptibility to disease, and pathology of the investigated hosts and is considered an essential component of early warning systems [7]. Using the passive health monitoring of roe deer in this study, we estimated that less than 1% of the total disease-related roe deer mortality in the country was analyzed. Each year, 13–52 animals were provided by hunters for laboratory analyses, but this number increased during the period of the study, which shows that the awareness of hunters in Slovenia is gradually increasing. Differences in the age and sex structure of carcasses and the (disease-related) total mortality indicated that the samples provided were not random, but mainly male and juvenile animals. It is likely that hunters more often provided samples that they considered more interesting: The animals showed unusual symptoms/behaviors and were, therefore, new to them, or they were more interesting because they concerned young males, which are potential trophy holders. As a result of the distortion of the samples provided as described above, the relevance of certain diseases to the mortality of the population is only given in general terms (in terms of size classes). Therefore, our monitoring cannot be considered an objective indicator of the specific disease-related mortality of roe deer in Slovenia. Nevertheless, the number of animals was high in absolute numbers, which is particularly important as clinical-pathological studies determine all disease processes affecting the animals studied [30,31]. We also suspect that animals with previously unknown external symptoms may be over-represented in our sample. We, therefore, think that such surveillance is reliable for monitoring the presence/absence of certain diseases (and other causes of mortality) in the population.

In recent years, only sporadic cases of the diseases listed at the OIE have been reported in roe deer in Europe. Here, we diagnosed several pathogens that cause sporadic infections in roe deer. No identified disease can be considered a significant threat to the health of other animals or humans, as was the case in previous studies conducted in Sweden [10] and Switzerland [7]. The literature generally describes the seroprevalence of antibodies against selected pathogens, whereas data describing the clinical course of roe deer diseases are rare and the only studies have been conducted in Switzerland [7], Sweden [10], and France [11].

In this study, infectious diseases were identified as the main cause of death more often (67%) than noninfectious diseases (28%). The results are consistent with the report from Switzerland [7], although the ratio between infectious and noninfectious diseases was higher in our case (in Switzerland, 46% and 39%, respectively). In contrast, noninfectious diseases were the main cause of death among roe deer in Sweden [10], with traumatic injuries (19%) and winter starvation (18%) topping of the list.

The low prevalence rate of 3.7% of neoplasia in roe deer found in this study is comparable to previous reports on roe deer [7,10]. The slightly higher prevalence reported here is due to a higher incidence of fibropapillomatosis compared with the report from Switzerland [7] and Sweden [10]. Fibropapillomas (68%) were the most frequently diagnosed tumor in Slovenia. Further data on fibropapillomatosis in deer in Slovenia were reported by Kmetec et al. [32]. In the present study, we confirmed the presence of PV by PCR from pronounced papillary structures collected from the various locations on the skin of six roe deer; in all cases (*n* = 13), fibropapillomas were confirmed by histological examination. PV infections of roe deer occur as an endemic infection in Hungary, Austria, Croatia [33], and Slovakia [34], whereas in Slovenia, they are more sporadic than endemic. Lymphosarcoma, the most frequently diagnosed tumor in roe deer in Switzerland [7], was diagnosed in only one case in our study. All other identified neoplasias (*n* = 5) were of different origins.

Here, parasite infections (48%) were the main cause of death in roe deer. All parasites identified here were common in roe deer and have also been recorded in other European countries [7,10,35,36]. The prevalence of parasite infections was three times higher than reported in Switzerland (12%) [7], France (10%) [11], and Sweden (11%) [10]. This might be due to the higher concentration of roe deer population in Slovenia (110,000 roe deer/20,271 km², which is 5.2 roe deer/km² [18,37] in relation to Switzerland (127,000 roe deer/41,285 km² (www.kora.ch (accessed on 29 December 2020)), which is 3 roe deer/km²), France (1,000,000 roe deer/640,679 km² [38], which is 0.6 roe deer/km²), and Sweden (250,000 roe deer/450,295 km² [39], which is 0.55 roe deer/km²). A probable explanation is the increased activity of wild ungulates grazing in areas shared with livestock, leading to the potential for transmission of parasitic nematodes between these groups [40]. Within the parasitic infections classified as the main cause of death, combined parasitosis with infestation of skin, gastrointestinal tract, and respiratory tract was fatal in 26% of cases, followed by infection with *Haemonchus contortus* (8.4%), *Chabertia ovina* (3.7%), lung parasites (Protostrongylidae) (3.3%), and *Dictyocaulus viviparus* (2.5%). In Switzerland, gastrointestinal nematodes were identified as the main cause of death in only 4% of cases [7], whereas in Sweden, verminous pneumonia was the most frequently reported parasitic disease [10]. In this study, different ectoparasites such as tick, louse, and hippoboscid fly species were identified in roe deer. The general prevalence of ectoparasite infestation, usually recorded as a secondary finding, was over 80%. The ectoparasite burden was high in most cases. This indicated that in addition to endoparasites, ectoparasites are also important in roe deer.

Bacterial infections were mostly associated with a mixed bacterial flora (4.5%), but *Trueperella pyogenes* was the most frequently diagnosed bacterium in our study (3%). In connection with the main disease, *T. pyogenes* was also the most frequently diagnosed bacterium in roe deer in Switzerland [7], whereas in France [11], *Pasteurella multocida* was the most frequently diagnosed bacterium. This result is not surprising, as it is assumed that *T. pyogenes* is the most widespread and most frequent opportunistic pathogen of the mucous membrane surfaces in domestic and wild animals [41,42]. *Escherichia coli* was also frequently associated with the main disease in Slovenia and Switzerland [7]. The foodborne pathogen Stx-harboring *E. coli* (STEC) is regularly detected in feces and carcasses of hunted wild ruminants, including roe deer, and can cause disease in humans [43,44]. The results of a study from Poland confirmed that roe deer are carriers of STEC/AE-STEC strains, which are potentially pathogenic to humans [44]. Other less common bacteria detected in the present study, such as *Staphylococcus aureus*, *P. multocida*, *Mannheimia granulomatis,* and *Yersinia pseudotuberculosis*, are consistent with an earlier report from Switzerland [7]. We report no cases of mycobacterial infections, but data from some European countries suggest that roe deer are susceptible to mycobacterial infections. *M. bovis*-induced pathology in roe deer has been reported in France [45] and England [46]. Antibodies against *M. avium paratuberculosis* (MAP) have been detected in Spain [47], Italy [48], and Norway [49], Austria [50], and Czech Republic [51] using PCR and cultivation methods. MAP-induced pathology has been detected in one case in Switzerland [7].

A lung infection with *Aspergillus fumigatus* was the cause of death in six animals; characteristic lesions of hypertrophic osteopathy (HO) were found in only one case. A low prevalence of this pathogen has been similarly reported in Switzerland [7] (three cases) and in France [11] (one case). The case of *A. fumigatus* causing mycotic pneumonia and secondary HO, also known as Marie’s disease, was described elsewhere [52]. Infections caused by *A. fumigatus* and characteristic lesions of HO have also been reported in roe deer in Germany [53] and Switzerland [7].

Trauma represents a significant proportion of roe deer deaths and is the main diagnosis of noninfectious origin. In this study, 13% of roe deer died due to trauma (including lightning strike), 48% of which were identified as blunt force trauma from traffic accidents and 25% were caused by firearms as the main diagnosis. In Sweden, trauma represented 19% of deaths [10], with predation, blunt force trauma from traffic accidents, and firearms being the main diagnoses, whereas in Switzerland, trauma represented 24% of deaths [7], with predation (9%) and blunt force trauma from traffic accidents (9%) being the main diagnoses. In France [11], trauma represented 28.5% of deaths in roe deer, with firearms (24%) and blunt force trauma from traffic accidents (13.7%) being the main diagnoses.

Metabolic disorders (9.8%) were the second most common cause of death in roe deer in Slovenia, including acidosis, bloating, and poisoning with oilseed rape (*Brassica napus*). The metabolic disorders in this study were consistent with those previously reported in roe deer in Switzerland [7] and partially consistent with those reported in Sweden [10].

Among the noninfectious causes of death in roe deer, we described the necropsy findings and histopathological examination of the tissue of two female deer found dead after a severe thunderstorm [54]. According to the scarce documentation in the literature, atmospheric lightning seems to be a rather unusual cause of death in wild animals. In most cases, the reports of electrocution injuries concern domesticated animals [55,56]. Although wild animals are more frequently exposed to this natural phenomenon, carcasses are usually not found.

The results of this research indicate a broad spectrum of roe deer diseases, but no identified disease can be considered a significant health threat to other wildlife species and/or to humans.

## 5. Conclusions

Roe deer can serve as a reservoir for many infectious agents, which promotes the spread and maintenance in the environment. Health surveillance and the monitoring of outbreaks of disease in wildlife can, therefore, be important for the welfare of wild species and for the protection of the health of domestic animals and humans. Although passive surveillance cannot be regarded as an objective indicator of specific disease-related mortality in roe deer, it is associated in absolute terms with a high number of animals, which is particularly important since clinical pathological studies determine all disease processes affecting the animals under study. This is the first comprehensive report on the causes of roe deer deaths in Slovenia, and provides an overview of the health status of roe deer during passive health monitoring. No identified disease in roe deer can be considered a significant threat to the health of other animals and/or humans in Slovenia.

## Figures and Tables

**Table 1:** Primary causes of mortality and morbidity in roe deer in Slovenia, 2000–2019.

Primary Disease Diagnosis	Number	%
Bacterial infections	75	14.8
Hernia	3	0.6
Lightning	2	0.4
Metabolic disorder	50	9.8
Mycotic infections	6	1.2
Miscellaneous	3	0.6
Multifactorial diseases	18	3.5
Neoplasia	19	3.7
Parasitic diseases	246	48.2
Trauma	64	12.5
Winter starvation	5	1
Indeterminable	19	3.7
Total	510	100

**Table 2:** Overview of the causes of death or emergency removal associated with the main disease diagnosed in roe deer in Slovenia, 2000–2019.

Cause of Death/Emergency Removal		Male	Female	Fawn	Yearling	Adult	Total	%
Noninfectious diseases								
	Hernia	2	1	2	1	-	3	0.6
	Lightning	-	2	1	1	-	2	0.4
	Metabolic disorder	28	22	12	24	14	50	9.8
	Miscellaneous	2	1	1	1	1	3	0.6
	Neoplasia	10	9	5	8	6	19	3.7
	Trauma	35	29	22	26	16	64	12.5
	Winter starvation	2	3	-	-	5	5	1
	Total	79	67	43	61	42	146	28.6
Infectious diseases								
	Bacterial							
	*Actinomyces* spp.	-	2	-	1	1	2	0.5
	*Bibersteinia trehalosi*	1	1	1	-	1	2	0.4
	*Clostridium perfringens*	2	2	-	2	2	4	0.8
	*Escherichia coli*	3	7	4	4	2	10	2
	*Listeria monocytogenes*	1	3	-	3	1	4	0.8
	*Mannheimia granulomatis*	1	1	1	1		2	0.4
	*Pasteurella multocida*	1	-	-	-	1	1	0.2
	*Pseudomonas* spp.	-	1	1	-	-	1	0.2
	*Serratia marcescens*	1	-	-	1	-	1	0.2
	*Staphylococcus aureus*	1	6	1	4	2	7	1.4
	*Trueperella pyogenes*	13	3	5	4	7	16	3
	*Yersinia pseudotuberculosis*	1	1	-	2	-	2	0.4
	Mixed bacterial flora	14	9	7	12	4	23	4.5
	Multifactorial diseases (bacteria/parasite)	6	12	8	5	5	18	3.5
	Mycotic							
	*Aspergillus fumigatus*	2	4	2	2	2	6	1.2
	Parasitic							
	*Chabertia ovina*	11	8	8	5	6	19	3.7
	*Dictyocaulus viviparus*	8	5	4	6	3	13	2.5
	*Fasciola hepatica*	2	2	2	1	1	4	0.8
	*Haemonchus contortus*	19	24	21	13	9	43	8.4
	*Hypoderma diana*	2	2	-	3	1	4	0.8
	Protostrongylidae	9	8	6	5	6	17	3.3
	Trichostrongylidae	4	5	2	2	5	9	1.8
	*Trichuris ovis*	1	3	-	1	3	4	0.8
	Coinfection	54	79	54	39	40	133	26
	Total	157	188	127	116	102	345	67.7
Indeterminable diseases		6	13	7	8	4	19	3.7
Total		242	268	177	185	148	510	100

## Data Availability

The data presented in this study are available on request from the corresponding author.
